# Combined Temperature Compensation Method for Closed-Loop Microelectromechanical System Capacitive Accelerometer

**DOI:** 10.3390/mi14081623

**Published:** 2023-08-17

**Authors:** Guowen Liu, Yu Liu, Zhaohan Li, Zhikang Ma, Xiao Ma, Xuefeng Wang, Xudong Zheng, Zhonghe Jin

**Affiliations:** 1 School of Aeronautics and Astronautics, Zhejiang University, Hangzhou 310058, China; 2 Beijing Institute of Aerospace Control Device, Beijing 100854, China

**Keywords:** MEMS accelerometer, combined compensation, voltage reference, temperature compensation

## Abstract

This article describes a closed-loop detection MEMS accelerometer for acceleration measurement. This paper analyzes the working principle of MEMS accelerometers in detail and explains the relationship between the accelerometer zero bias, scale factor and voltage reference. Therefore, a combined compensation method is designed via reference voltage source compensation and terminal temperature compensation of the accelerometer, which comprehensively improves the performance over a wide temperature range of the accelerometer. The experiment results show that the initial range is reduced from 3679 ppm to 221 ppm with reference voltage source compensation, zero-bias stability of the accelerometer over temperature is increased by 14.3% on average and the scale factor stability over temperature is increased by 88.2% on average. After combined compensation, one accelerometer zero-bias stability over temperature was reduced to 40 μg and the scale factor stability over temperature was reduced to 16 ppm, the average value of the zero-bias stability over temperature was reduced from 1764 μg to 36 μg, the average value of the scale factor stability over temperature was reduced from 2270 ppm to 25 ppm, the average stability of the zero bias was increased by 97.96% and the average stability of the scale factor was increased by 98.90%.

## 1. Introduction

An accelerometer is a typical inertial sensor, which has a wide range of important applications in aviation, navigation, aerospace, weapons and civilian fields. However, the large size and high price of traditional accelerometers limit their application. With the development of MEMS (microelectromechanical system) technology, a variety of MEMS accelerometers have emerged, and their small size, small power consumption and wide application range have aroused the interest of research from all walks of life. At present, the sensitive structure of high-performance accelerometers mostly uses an all-silicon structure, which has developed rapidly due to its advantages of full-temperature performance, such as Safran’s Colibrys sandwich all-silicon accelerometer with a zero-bias stability of 30 μg in 2020 [1]. As a typical representative, the Litton SiAC^TM^ silicon accelerometer has a range of more than 100 g, a zero bias better than 20 μg and a scale factor stability better than 50 ppm. In addition to the improvement of sensitive structures, there are some improvements in the circuit to enhance the accuracy of MEMS accelerometers. A 2012 Colibrys article introduced a navigation-grade Sigma-Delta MEMS accelerometer [2]. The accelerometer interface used a preamplifier and an ADC (analog-to-digital conversion) in part, and the rest of the circuitry was carried out digitally. At the same time, the closed-loop structure is adopted to reduce the equivalent noise and quantization noise of the structure, improve the linearity of the structure and ensure performance within the vibration environment.

Multiple ways have been proposed to improve the thermal behavior of MEMS accelerometers. Some studies propose the structure in [3,4,5,6,7], other studies reduce the thermal drift via compensating circuits and algorithm [8,9,10,11,12,13,14,15,16,17,18,19]. In 2015, Sergei A. Zotov et al. introduced a high-quality-factor resonant MEMS accelerometer [3]. To address drift over temperature, the MEMS sensor die incorporates two identical tuning forks with opposing axes of sensitivity. Demodulation of the differential FM output from the two simultaneously operated oscillators eliminates common mode errors and provides an FM output with continuous thermal compensation. Allan deviation of the differential FM accelerometer revealed a bias instability of 6 μg at 20 s, along with an elimination of any temperature drift due to increases in averaging time. In 2018, Giuseppe Ruzza et al. introduced the thermal compensation of low-cost MEMS accelerometers for tilt measurements [4], which have developed a miniaturized thermal chamber mounted on a tilting device to account for tilt angle variation. It can be determined whether it is warming or cooling the cycles, then select the corresponding compensation equation. After compensation, the RMS errors calculated for both the x- and y-axes decreased by 96%, but increased the complexity of the technique. In 2018, Wei Xu et al. reported a dual-differential accelerometer with an all-silicon structure with 3-times improved full temperature stability [5]. In 2020, Niu H et al. reported a comb accelerometer made of an all-silicon structure with a zero-bias stability of 100 μg [6]. In 2021, Liu Dandan et al. introduced an in situ compensation method for the scale factor temperature coefficient of a single-axis force-balanced MEMS accelerometer [7], which integrates the thermistor with the accelerometer to detect the temperature change of the accelerometer in real time, compensates for the change of the scale factor and reduces the temperature coefficient in the range of 25 °C to 50 °C for 6 ppm/°C.

In 2015, Qingjiang Wang et al. introduced the thermal characteristics of typical microelectromechanical system (MEMS) inertial measurement units (IMUs) with a reliable thermal test procedure [8]. The first-order piecewise function is introduced to establish the thermal models. The performance of both IMUs and inertial navigation systems improved significantly after compensation with the established thermal models. In 2019, Qing Lu et al. introduced a fusion algorithm-based temperature compensation method for a high-g MEMS accelerometer [10], which combines empirical mode decomposition (EMD), wavelet thresholding and temperature compensation to process measurement data from a high-g MEMS accelerometer. The experimental data show that the acceleration random walk changes from 1712.66 g/h/Hz^0.5^ to 79.15 g/h/Hz^0.5^ and the zero-deviation stability changes from 49,275 g/h to 774.7 g/h. In 2020, Vasco L et al. introduced a small-size, vacuum-packaged capacitive MEMS accelerometer through Sigma-Delta modulation [11], using an FPGA (field-programmable gate array) to achieve three different modulation levels, allowing for the flexible real-time adjustment of loop parameters. In 2021, Javier Martínez et al. introduced a lightweight thermal compensation technique for a MEMS capacitive accelerometer [12,13]. In this work, a light calibration method based on theoretical studies was proposed to obtain two characteristic parameters of the sensor’s operation: the temperature drift of the bias and the temperature drift of the scale factor. This method requires less data to obtain the characteristic parameters, allowing for faster calibration. In 2021, Pengcheng Cai et al. introduced an improved difference temperature compensation method for MEMS resonant accelerometers [14], which proposed an improved temperature compensation approach, called proportional difference, for accelerometers based on differential frequency modulation. Experiment results demonstrate that the temperature sensitivity of the prototype sensor was reduced from 43.16 ppm/°C to 0.83 ppm/°C within the temperature range of −10 °C to 70 °C using the proposed method. In 2022, Qi Bing et al. introduced a novel accurate temperature drift error estimation model using microstructural thermal analysis [15], which obtains the complete temperature correlated quantities through structural thermal deformation. Moreover, the particle swarm optimization genetic algorithm back propagation neural network [16] was used to improve the accuracy and real-time recognition of accelerometer models. Compared with the traditional model, the accuracy was improved by 16%, and the number of iterations reduced by up to 99.86%. In 2023, Gangqiang Guo et al. introduced temperature drift compensation of a MEMS accelerometer based on DLSTM and ISSA [17], which improved bias instability, rate random walk and rate ramp with an increase of 96.68% on average. In 2023, Bo Yuan et al. presented a calibration and thermal compensation method for triaxial accelerometers based on the Levenberg–Marquardt (LM) algorithm and polynomial methods [18]. Within the temperature range of −40 °C to 60 °C, the temperature drifts of x- and y-axes reduced from −13.2 and 11.8 mg to −0.9 and −1.1 mg, respectively. The z-axis temperature drift was reduced from −17.9 to 1.8 mg. In 2023, Mingkang Li et al. reported an approach of in-operation temperature bias drift compensation based on phase-based calibration for a stiffness-tunable MEMS accelerometer with double-sided parallel plate (DSPP) capacitors [19]. The demodulated phase of the excited response exhibits a monotonic relationship with the effective stiffness of the accelerometer. Through the proposed online compensation approach, the temperature drift of the effective stiffness can be detected through the demodulated phase and compensated in real time by adjusting the stiffness-tuning voltage of DSPP capacitors. The temperature drift coefficient (TDC) of the accelerometer is reduced from 0.54 to 0.29 mg/°C, and the Allan variance bias instability of about 2.8 μg is not adversely affected.

Due to its excellent characteristics, all-silicon accelerometers can be used in navigation and guidance fields, such as the navigation and control of small UAVs, short-range tactical weapon guidance, etc. This article analyzes the operating principle of capacitive accelerometers and focuses on the factors that affect the full-temperature performance of capacitive accelerometers. In this paper, an all-silicon comb accelerometer with anchor zone stress cancellation technology will be analyzed [20], and a combined compensation method is designed to improve the full-temperature performance of the accelerometer via reference voltage source compensation and terminal temperature compensation of the accelerometer, which provides a basis for the development of capacitive accelerometers with a high range, high precision and high sensitivity.

## 2. MEMS Accelerometer Composition

**Figure 1:**
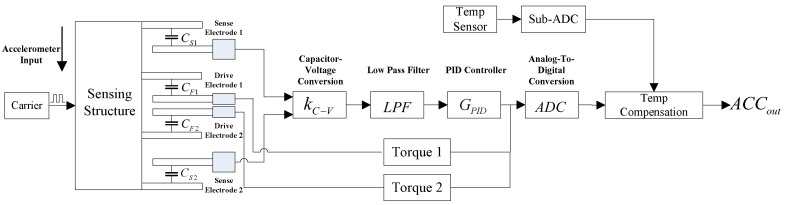
Block diagram of a MEMS accelerometer.

**Figure 2:**
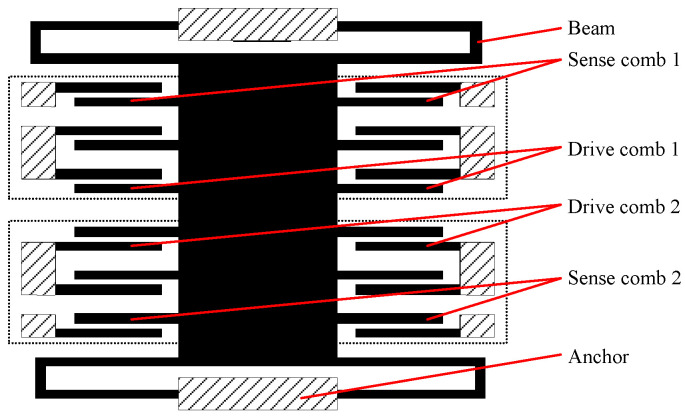
Schematic diagram of a MEMS accelerometer-sensitive structure.

As shown in Figure 1, the MEMS accelerometer includes and the accelerometer-sensitive structure which shown in Figure 2, capacitor–voltage conversion module, low-pass filter, PID controller, torque meter, temperature sensor, analog-to-digital conversion module and temperature compensation algorithm module [21,22].

## 3. MEMS Accelerometer Principle

As shown in Figure 1 above, when the external acceleration input occurs, the sensitive structure of the accelerometer is displaced relative to the carrier coordinate system. The distance between the sense electrode 1 and the sensing structure increases, so that the sense capacitance C_S1_ decreases, and the sense electrode 2 decreases with the sensitive structure comb electrode, so that the sense capacitance C_S2_ increases.

The sense capacitance C_S1_ and the sense capacitance C_S2_ convert the two capacitor values into two voltage values through the capacitance–voltage conversion circuit, and obtain the differential voltage via differential operation. This differential voltage is output to the analog-to-digital conversion module through a low-pass filter and PID controller. At the same time, the output of the PID controller is amplified by torque 1 and torque 2, and then feed back to the drive electrode 1 and 2, respectively. Drive electrode 1 forms drive capacitance with the sensing structure electrode C_F1_, and drive electrode 2 forms drive capacitance with sensing structure electrode C_F2_. Because the torque applied to the two drive voltages differs, the electrostatic attraction of C_F1_ is greater than that of C_F2_, ultimately forcing the accelerometer-sensitive structure to remain near the initial position at all times. The output voltage of the PID controller is fed into the analog-to-digital conversion module and converted into a digital signal, and finally, the digital output of the temperature sensor performs temperature compensation calculations and outputs the final acceleration signal.

When the electrostatic force is equilibrated, the position of the movable comb (the electrical zero point) deviates from the mechanical zero point, *δ*. For the movable comb, the electrostatic force balance equation is:

(1) $$K_{m}\delta +F_{e}+ma=0$$

In this equation, *K_m_* is the mechanical stiffness of the accelerometer cantilever beam, *F_e_* is the electrostatic force, *m* is the mass of the accelerometer sensitive structure and *a* is the acceleration input.

According to the static equilibrium analysis of the accelerometer closed-loop system [22], the output when the electrostatic force equilibrium is obtained is:

(2) $$V_{fb}=\frac{V_{R}\left(\frac{d_{0}}{\delta }+\frac{\delta }{d_{0}}\right)}{2K_{fb}}\pm \frac{V_{R}\left(\frac{d_{0}}{\delta }-\frac{\delta }{d_{0}}\right)}{2K_{fb}}\sqrt{1+\frac{2K_{m}}{C_{f}}{\left(\frac{\delta }{V_{R}}\right)}^{2}+\frac{2ma\delta }{C_{f}V_{R}^{2}}}$$

Which can be expanded to:

(3) $$V_{fb}=\frac{\delta V_{R}}{d_{0}K_{fb}}-\frac{\left(K_{m}\delta +ma\right)d_{0}}{2C_{f}V_{R}K_{fb}}$$

In this equation, *C_f_* is the sense capacitance, *V_R_* is the driving reference voltage, *K_m_* is the mechanical stiffness of the accelerometer cantilever beam, *V_fb_* is the feedback output, *K_fb_* is the feedback coefficient, *m* is the mass of the accelerometer sensitive structure, *a* is the acceleration input, *d*_0_ is the comb gap and *F_e_* is the electrostatic force.

Equation (3) provides a quadratic model of the static equilibrium state, so a zero-bias *K*_0_ is:

(4) $$K_{0}=\frac{\delta V_{R}}{d_{0}K_{fb}}-\frac{K_{m}\delta d_{0}}{2C_{f}V_{R}K_{fb}}$$

The primary term coefficient *K*_1_ is:

(5) $$K_{1}=-\frac{md_{0}}{2C_{f}V_{R}K_{fb}}$$

**Figure 3:**
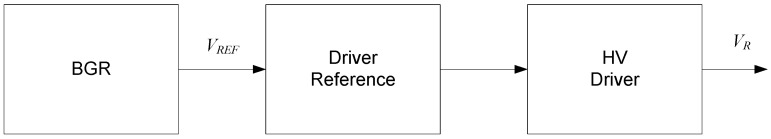
Accelerometer ASIC reference voltage source.

As seen from Equations (4) and (5), the zero bias and scale factor are all related to the drive reference voltage *V_R_*. From this, the temperature characteristics have a direct impact on the performance of the circuit. Figure 3 shows the flow direction of the main reference voltage of the closed-loop control circuit. It can be confirmed that the band-gap reference (BGR) used by the ASIC is the final source of the subsequent drive reference voltage, so its temperature characteristics have a direct impact on the temperature characteristics of the entire accelerometer.

## 4. Temperature Compensation Method

### 4.1. Reference Voltage Source Compensation

**Figure 4:**
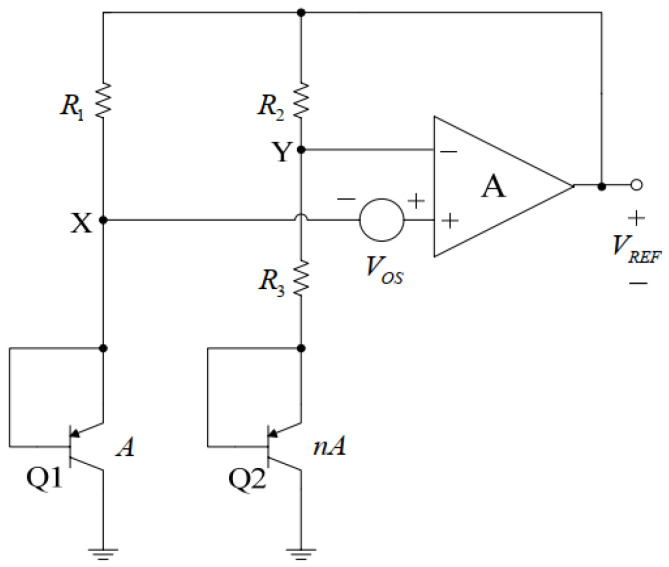
Band-gap reference source schematic.

A common band-gap reference circuit [23,24] is based on the principle of adding two voltages of equal magnitude and opposite temperature coefficients to obtain a temperature-independent voltage. The negative temperature coefficient voltage is realized through the base-emitter voltage, *V_BE_*, of the substrate tertiary transistor. The positive temperature coefficient is accomplished through the base-emitter voltage difference, Δ*V_BE_*, of two transistors operating at different current densities. In practice, the sum of the two temperature coefficients is not exactly zero. A typical band-gap reference is shown in the Figure 4 below.

In the Figure 4, *V_OS_* is the offset voltage and the emitter area of bipolar transistor Q2 is n times that of Q1. Under the action of the op amp, the voltages of nodes X and Y are equal, the current flowing through bipolar transistors Q2 and Q1 is also equal and the base-emitter voltage difference between Q1 and Q2 is:

(6) $$\Delta V_{BE}=V_{T}\ln n$$

*V_T_* = *kT*/*q*, *k* is the Boltzmann constant, *T* is the absolute temperature and *q* is the electron charge. *V_BE2_* is a negative temperature coefficient. The reference voltage output with the offset in this case is [23]:

(7) $$V_{REF}=V_{BE2}+(1+\frac{R_{2}}{R_{3}})(V_{T}\ln n+V_{OS})$$

By selecting the appropriate ratio of *n* to resistors *R*_2_ and *R*_3_, a temperature-independent output reference voltage can be obtained. In integrated circuit design, for symmetry considerations, *n* is generally taken as 8, and *R*_2_ and *R*_3_ are implemented through the resistor repair network and controlled via digital registers or other means.

**Figure 5:**
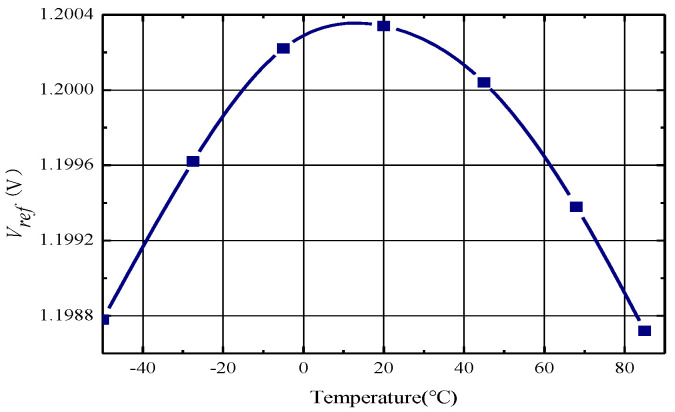
Simulation of temperature characteristics of band-gap references.

The Figure 5 shows the simulation results of the temperature characteristics of the output voltage. The maximum change in output voltage is 1.55 mV and the temperature coefficient is 9 ppm/°C, from −50 °C to 85 °C.

The number of transistors in the ASIC design process is a definite value. The temperature characteristics are determined by the acceleration sensor itself and are affected by the processing process. Thus, the resistor can be adjusted later by forming a resistor network.

According to Equation (5), the scale factor of the system is directly related to the high drive voltage, VR, so the temperature characteristics of the scale factor will be directly affected by the characteristics of the high drive voltage and therefore also by the temperature characteristics of the band-gap reference.

Limited by the ASIC chip pin count and anti-interference design, the direct output of the band-gap reference is not easy to measure, so we selected the 4.5 V output point of the chip to evaluate the temperature characteristics of the reference.

**Figure 6:**
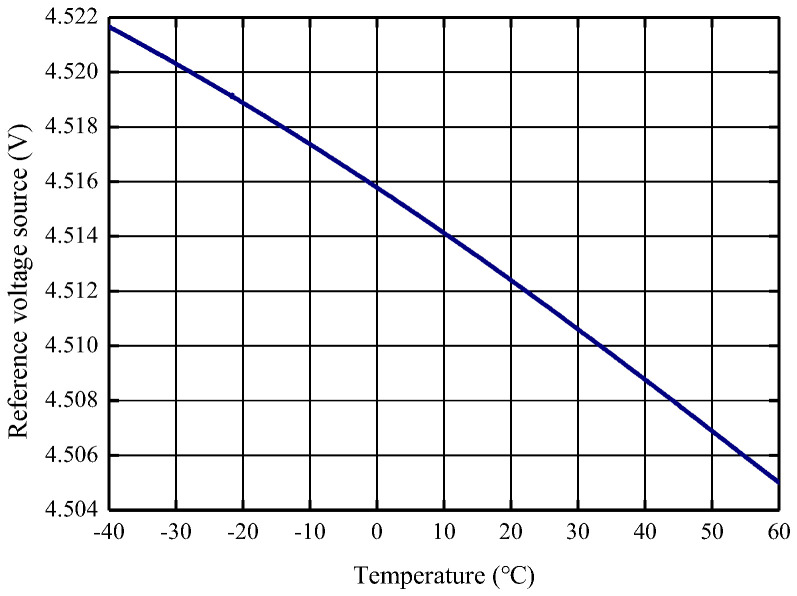
Factory state reference temperature characteristics of the circuit chip.

**Figure 7:**
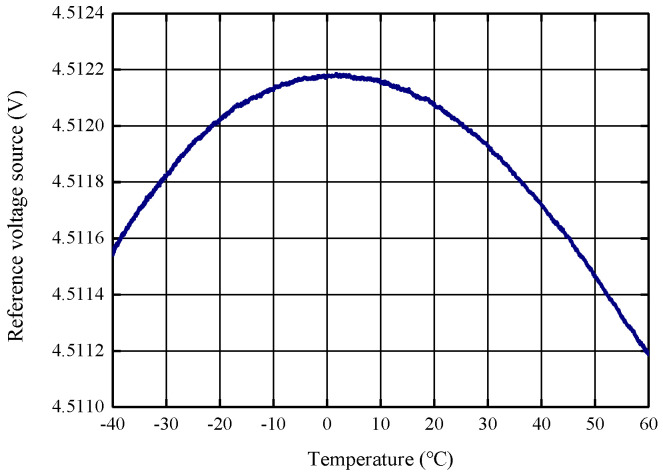
Modified reference temperature characteristics of the circuit chip.

Due to the deviation between the ASIC design value and the actual processing, the actual measured temperature characteristics of the 4.5 V reference voltage are shown in Figure 6 below, with an initial range of 3679 ppm, and after adjusting the resistor network through multiple rounds of iterative experiments, the final range is 221 ppm, as shown in Figure 7.

### 4.2. Accelerometer Terminal Temperature Compensation

In order to further improve the performance over a wide temperature range of the accelerometer, the terminal third-order temperature compensation of the accelerometer is continued on the basis of the reference voltage source compensation to improve the performance over a wide temperature range of the accelerometer. Through the stability-over-temperature modeling experiment of the accelerometer, the temperature sensor output, *T_i_*, zero-bias *K*_0*i*_ and scale factor, *K*_1*i*_, of the accelerometer at each temperature point are obtained. The third-order polynomial model is fit to the zero bias vs. accelerometer temperature sensor output, where *p*_0_, *p*_1_, *p*_2_ and *p*_3_ are the 0–3-order coefficients of the model. Then, the accelerometer zero bias *K*_0_ is:

(8) $$K_{0}=p_{3}T^{3}+p_{2}T^{2}+p_{1}T+p_{0}$$

The fitting error *E* is:

(9) $$E=\sum_{i=0}^{n}{\left[K_{0}-\left(p_{3}T_{i}^{3}+p_{3}T_{i}^{2}+p_{1}T_{i}+p_{0}\right)\right]}^{2}$$

In order to minimize the fitting error, it is necessary to make its various deviations $\frac{\partial E}{\partial T_{i}}=0$

$$-2\sum_{i=0}^{n}\left[K_{0}-\left(+p_{3}T_{i}^{3}+p_{3}T_{i}^{2}+p_{1}T_{i}+p_{0}\right)\right]=0$$

(10) $$-2\sum_{i=0}^{n}\left[K_{0}-\left(p_{3}T_{i}^{3}+p_{3}T_{i}^{2}+p_{1}T_{i}+p_{0}\right)\right]\cdot T_{i}^{1}=0$$

$$-2\sum_{i=0}^{n}\left[K_{0}-\left(p_{3}T_{i}^{3}+p_{3}T_{i}^{2}+p_{1}T_{i}+p_{0}\right)\right]\cdot T_{i}^{2}=0$$

$$-2\sum_{i=0}^{n}\left[K_{0}-\left(p_{3}T_{i}^{3}+p_{3}T_{i}^{2}+p_{1}T_{i}+p_{0}\right)\right]\cdot T_{i}^{3}=0$$

Sorted out, it is:

(11) $$\left[\begin{array}{cccc} n & \sum_{i=0}^{n}T_{i} & \sum_{i=0}^{n}T_{i}^{2} & \sum_{i=0}^{n}T_{i}^{3}\\ \sum_{i=0}^{n}T_{i} & \sum_{i=0}^{n}T_{i}^{2} & \sum_{i=0}^{n}T_{i}^{3} & \sum_{i=0}^{n}T_{i}^{4}\\ \sum_{i=0}^{n}T_{i}^{2} & \sum_{i=0}^{n}T_{i}^{3} & \sum_{i=0}^{n}T_{i}^{4} & \sum_{i=0}^{n}T_{i}^{5}\\ \sum_{i=0}^{n}T_{i}^{3} & \sum_{i=0}^{n}T_{i}^{4} & \sum_{i=0}^{n}T_{i}^{5} & \sum_{i=0}^{n}T_{i}^{6} \end{array}\right]\cdot \left[\begin{array}{c} p_{0}\\ p_{1}\\ p_{2}\\ p_{3} \end{array}\right]=\left[\begin{array}{c} \sum_{i=0}^{n}K_{0_{i}}\\ \sum_{i=0}^{n}K_{0_{i}}T_{i}\\ \sum_{i=0}^{n}K_{0_{i}}T_{i}^{2}\\ \sum_{i=0}^{n}K_{0_{i}}T_{i}^{3} \end{array}\right]$$

Let the van der Mond matrix *V*:

(12) $$V=\left[\begin{array}{cccc} 1 & 1 & \ldots & 1\\ T_{1} & T_{2} & \ldots & T_{n}\\ T_{1}^{2} & T_{2}^{2} & \ldots & T_{n}^{2}\\ T_{1}^{3} & T_{2}^{3} & \ldots & T_{n}^{3} \end{array}\right]$$

Sorted out, it is:

(13) $$VV^{T}=\left[\begin{array}{cccc} n & \sum_{i=0}^{n}T_{i} & \sum_{i=0}^{n}T_{i}^{2} & \sum_{i=0}^{n}T_{i}^{3}\\ \sum_{i=0}^{n}T_{i} & \sum_{i=0}^{n}T_{i}^{2} & \sum_{i=0}^{n}T_{i}^{3} & \sum_{i=0}^{n}T_{i}^{4}\\ \sum_{i=0}^{n}T_{i}^{2} & \sum_{i=0}^{n}T_{i}^{3} & \sum_{i=0}^{n}T_{i}^{4} & \sum_{i=0}^{n}T_{i}^{5}\\ \sum_{i=0}^{n}T_{i}^{3} & \sum_{i=0}^{n}T_{i}^{4} & \sum_{i=0}^{n}T_{i}^{5} & \sum_{i=0}^{n}T_{i}^{6} \end{array}\right],\;V\left[\begin{array}{c} K_{0_{1}}\\ K_{0_{2}}\\ \vdots \\ K_{0_{n}} \end{array}\right]=\left[\begin{array}{c} \sum_{i=0}^{n}K_{0_{i}}\\ \sum_{i=0}^{n}K_{0_{i}}T_{i}\\ \sum_{i=0}^{n}K_{0_{i}}T_{i}^{2}\\ \sum_{i=0}^{n}K_{0_{i}}T_{i}^{3} \end{array}\right]$$

Therefore:

(14) $$VV^{T}\cdot \left[\begin{array}{c} p_{0}\\ p_{1}\\ p_{2}\\ p_{3} \end{array}\right]=V\cdot \left[\begin{array}{c} K_{0_{1}}\\ K_{0_{2}}\\ \vdots \\ K_{0_{n}} \end{array}\right]$$

Finally, the third-order fitting coefficient of the zero bias vs. accelerometer temperature sensor output is obtained:

(15) $$\left[\begin{array}{c} p_{0}\\ p_{1}\\ p_{2}\\ p_{3} \end{array}\right]={\left(VV^{T}\right)}^{-1}V\cdot K_{0}$$

Similarly, a third-order polynomial model of the scale factor change vs. the output of the accelerometer temperature sensor can be obtained:

(16) $$\frac{{K_{1}}^{\prime }}{K_{1_{T}}}=q_{3}T^{3}+q_{2}T^{2}+q_{1}T+q_{0}$$

${K_{1}}^{\prime }$ is the scale factor of the accelerometer at room temperature, $K_{1_{T}}$ is the scale factor of the accelerometer at each temperature point and *q*_0_, *q*_1_, *q*_2_ and *q*_3_ are the 0–3-coefficients of the model.

The ASIC chip is known to read the temperature sensor data, *T*, and the accelerometer output measurement, *D_a_*. According to Equations (8) and (16), the accelerometer temperature compensation value *D_out_* is:

(17) $$D_{out}=\left(D_{a}-K_{0}\right)\cdot \left(\frac{{K_{1}}^{\prime }}{K_{1_{T}}}\right)$$

## 5. Comparative Experiments

### 5.1. Uncompensated Experiments

**Figure 8:**
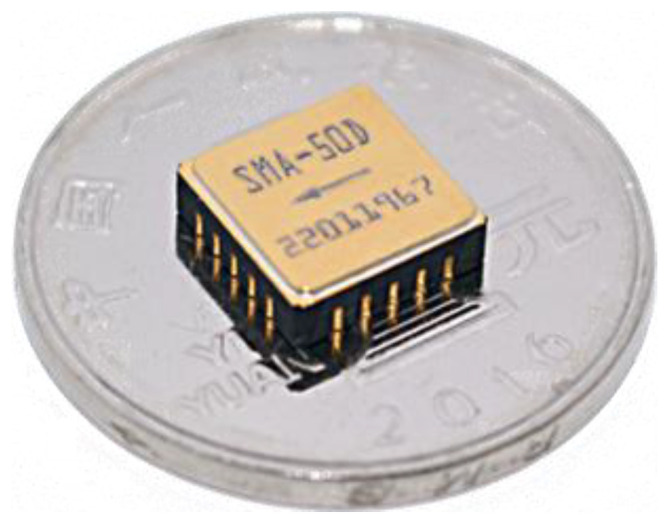
Accelerometer product photo.

**Figure 9:**
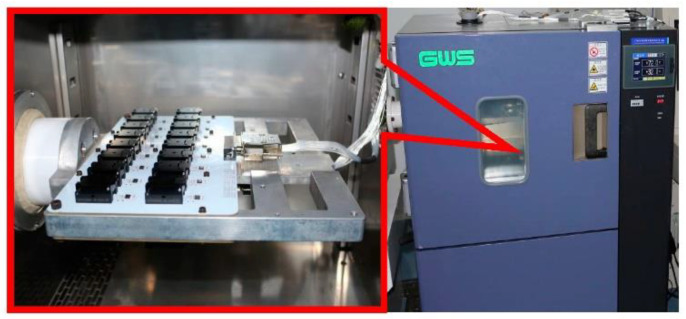
Accelerometer temperature performance test system.

**Figure 10:**
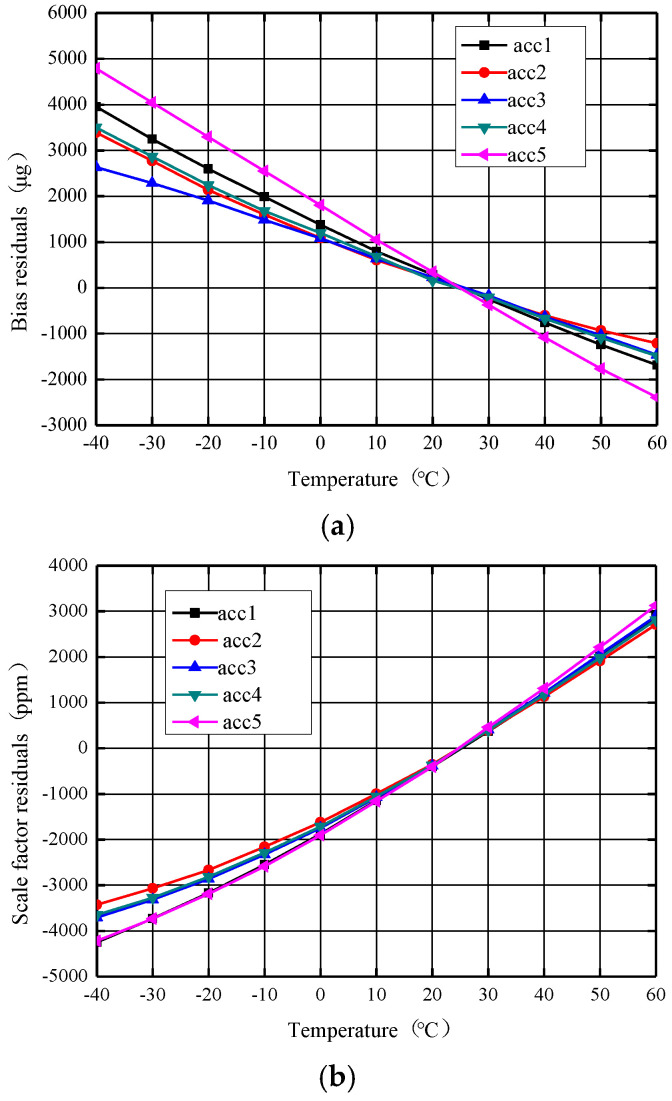
The reference voltage source is uncompensated for the zero bias and scale factor vs. temperature. (**a**) Zero bias curve against temperature. (**b**) Scale factor curve against temperature.

Temperature tests have been performed for the −40 °C to +60 °C range with a 1 °C/min temperature gradient. The test data collection is smoothed for 1 s, the bias and scale factor are calculated using the range and the stability is calculated using the standard deviation. Figure 8 shows the accelerometer product photo, where the mass is 0.7 g and size 9 × 9 × 2.7 mm^3^. Figure 9 shows the accelerometer temperature performance test system, model GWS EG-02JAS. The temperature uncompensated accelerometer’s zero bias and scale factor as a function of temperature are shown in Figure 10 below. From Figure 10a, it can be seen that the change (peak–peak) of the zero bias of the five MEMS accelerometers with temperature compensation from −40 °C to +60 °C is distributed between 4098 μg and 7183 μg, and the zero-bias stability (1σ) over a wide temperature range is distributed between 1374 μg and 2400 μg. The result of each accelerometer varies linearly with temperature. From Figure 10b, it can be seen that the temperature variation (peak–peak) of the scale factor of the five MEMS accelerometers is distributed between 6135 ppm and 7347 ppm, and the stability of the scale factor over a wide temperature range (1σ) is distributed between 2075 ppm and 2472 ppm. The scale factor of each accelerometer is also basically linear against temperature.

### 5.2. Accelerometer Stability over Temperature Experiment after Independent Reference Voltage Source Compensation

**Figure 11:**
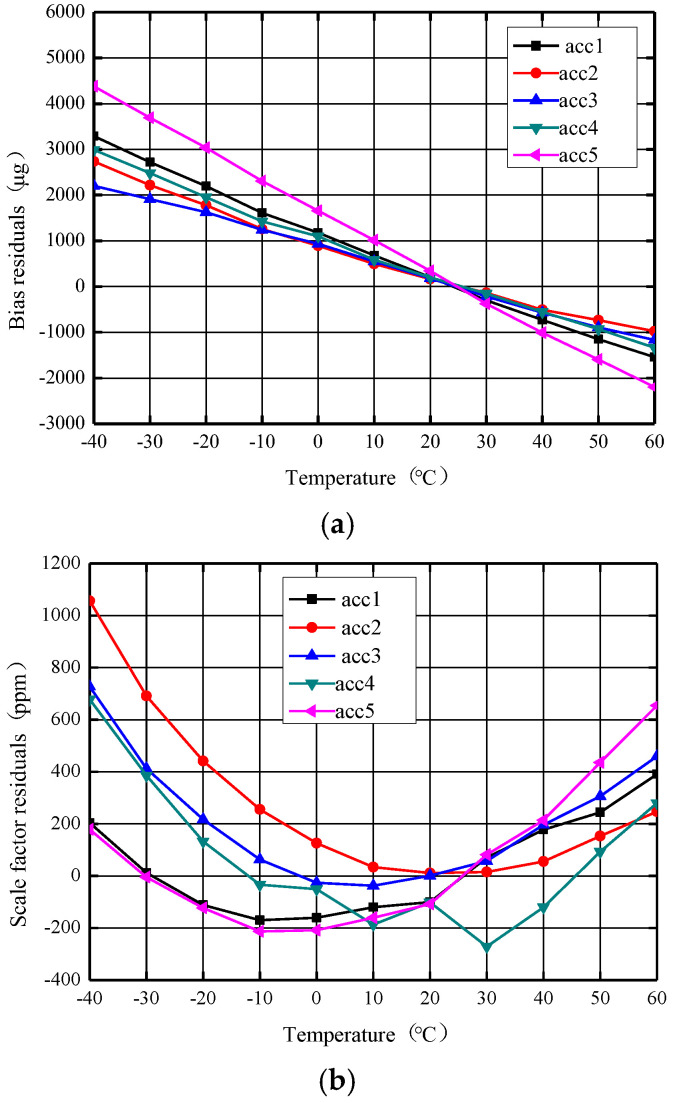
The zero bias and scale factor curves against temperature after reference voltage source compensation. (**a**) Zero bias curve against temperature. (**b**) Scale factor curve against temperature.

The zero bias and scale factor of the accelerometer with reference voltage source compensation as a function of temperature are shown in Figure 11. As seen in Figure 11a, after the reference voltage source compensation, the zero bias of the five MEMS accelerometers was distributed between 3367 μg and 6576 μg with the temperature from −40 °C to +60 °C, and the zero bias stability over temperature (1σ) was distributed between 1156 μg and 2196 μg, and the zero bias stability of each accelerometer increased by an average of 14.3%, with the result remaining linear against temperature. As seen from Figure 11b, the temperature variation (peak–peak) of the scale factor of the five MEMS accelerometers was distributed between 564 ppm and 1044 ppm, the stability of the scale factor over temperature (1σ) was distributed between 191 ppm and 330 ppm and the stability of scale factor of each accelerometer was increased by 88.2% on average. The comparative experiment results show that the accelerometer scale factor performance over temperature can be significantly improved by compensating the reference source temperature. The scale factor of each accelerometer as a function of temperature also changes from a basic linear curve to a second-order curve.

### 5.3. Accelerometer Stability over Temperature Experiment after Independent Terminal Temperature Compensation

**Figure 12:**
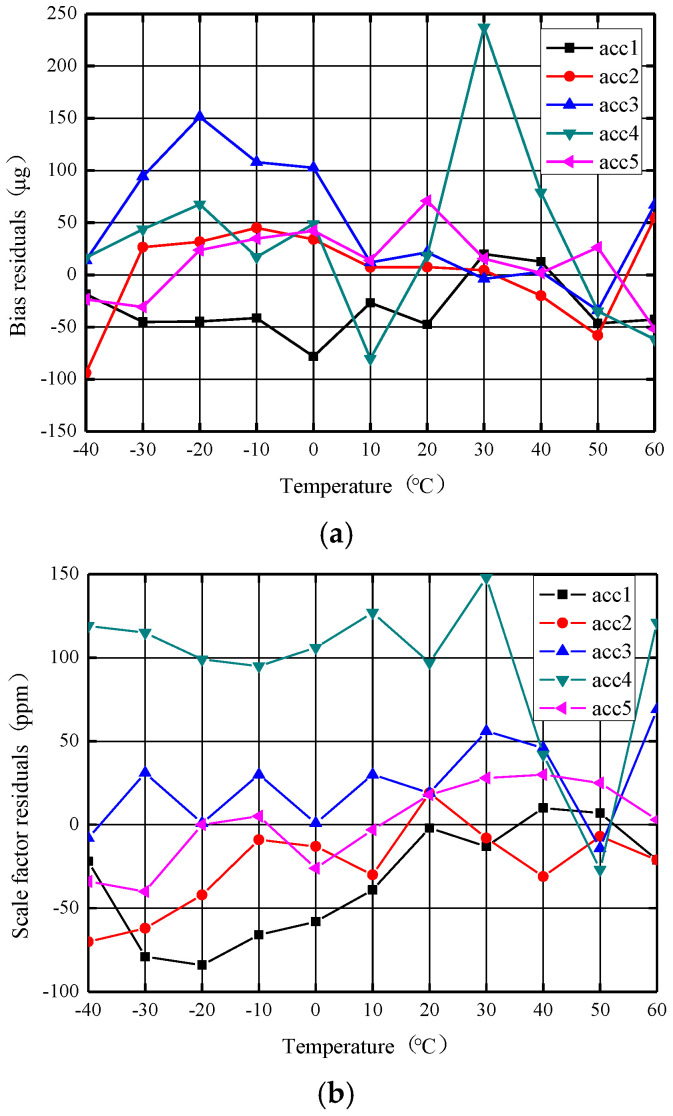
The zero bias and scale factor curve with temperature after terminal temperature compensation. (**a**) Zero bias curve against temperature. (**b**) Scale factor curve against temperature.

To verify the effectiveness of the combined compensation method, the stability performance of the accelerometer after terminal temperature compensation was continued to be verified. Using the same five accelerometers and terminal temperature compensation, a plot of the accelerometer zero bias and scale factor as a function of temperature is obtained. Figure 12a shows that in the range of −40–+60 °C, the zero bias total temperature variation (peak–peak) of the five accelerometers is distributed in 83–317 μg, and the zero bias stability over temperature (1σ) is distributed in 26–85 μg. In the same temperature range, the total temperature variation (peak–peak) of the scale factor of the five accelerometers shown in Figure 12b is distributed in 71–175 ppm, and the stability of the scale factor over temperature (1σ) is distributed within 25–48 ppm. Among them, one accelerometer fluctuation is obviously more severe than the others, mainly because the processed accelerometer still has poor consistency and some degree of discreteness, but the overall results are still within the range. Terminal temperature compensation significantly improves accelerometer bias and scale factor performance over a wide temperature range.

### 5.4. Stability over Temperature Experiment with Combined Temperature Compensation

**Figure 13:**
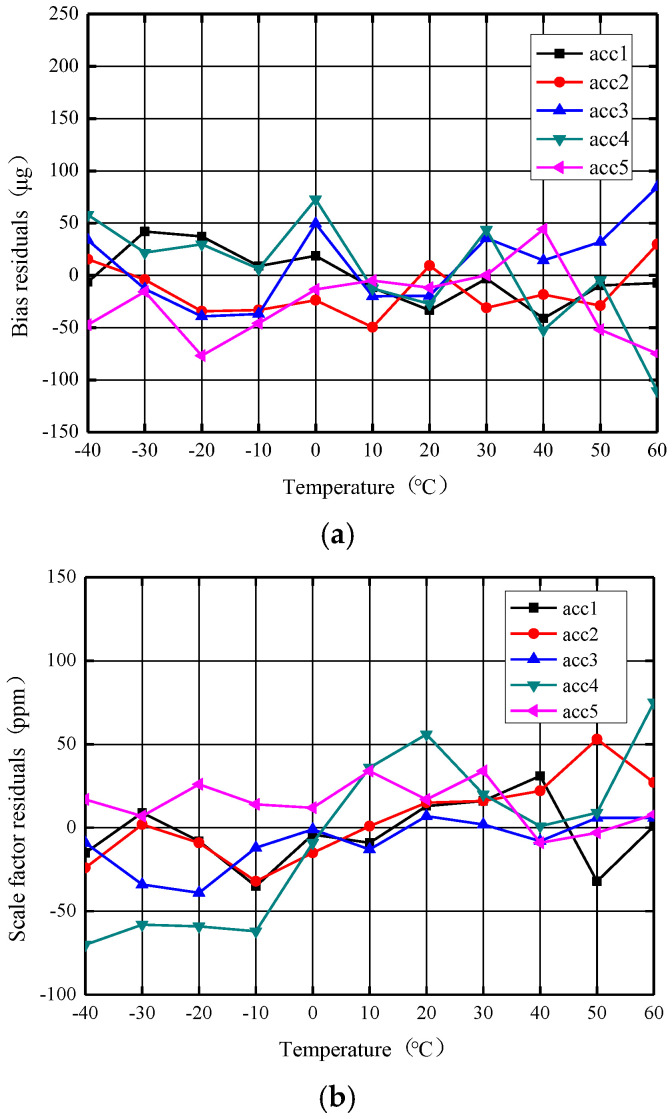
The zero bias and scale factor curve with temperature after the combined compensation. (**a**) Zero bias curve against temperature. (**b**) Scale factor curve against temperature.

The combined compensation of reference voltage source compensation and end-temperature compensation was used for the same five accelerometers to verify its validity. The zero bias and scale factor of the accelerometers as a function of temperature is shown in Figure 13. Figure 13a shows that in the range of −40–+60 °C, the zero bias temperature variation (peak–peak) of the five accelerometers was distributed within 79–183 μg, and the zero-bias stability over temperature (1σ) was distributed within 25–53 μg. Compared with only terminal temperature compensation, the combined compensation at the five accelerometers has improved the zero-bias stability at a wide temperature range by an average of 24.5%. Through combined temperature compensation, the total temperature variation (peak–peak) of the scale factor of the five accelerometers shown in Figure 13b was distributed within 43–145 ppm, and the stability of the scale factor over temperature (1σ) was distributed within 14–51 ppm. The scale factor stability of the five accelerometers was improved by an average of 25.5% compared to using only terminal temperature compensation. This combined compensation method comprehensively improves the accelerometer performance over a wide temperature range and proves the effectiveness of this method.

Table 1 shows the zero bias and scale factor full temperature experiment data for an accelerometer numbered ACC3 before and after various compensations. Combined compensation has obvious advantages over any single compensation method.

## 6. Conclusions

MEMS accelerometers are characterized by a small size, light weight and low cost. However, there is also a negative effect of the drift in temperature coefficient of the reference voltage source. This effect creates errors when the accelerometer operates at a wide temperature range, ultimately affecting the output signal of the accelerometer. In order to reduce the influence of the temperature coefficient of the reference voltage source, a method of temperature compensation of the reference voltage source is proposed, which is to reduce the influence of the temperature drift from the reference voltage source. To further improve the accelerometer performance over a wide temperature range, the accelerometer is compensated for the terminal third-order temperature compensation and the reference voltage source compensation. The experiment results show that the average value of zero-bias stability of the accelerometer with combined temperature compensation of reference voltage source compensation and terminal temperature compensation is reduced from 1764 μg to 36 μg, and the average stability of the scale factor over temperature is reduced from 2270 ppm to 25 ppm. The zero-bias stability over temperature is improved by 97.96% on average. The scale factor stability over temperature is improved by 98.90% on average. The combined compensation method greatly improves the accelerometer’s performance over a wide temperature range, which increases the accelerometer’s high-precision application capability. The accelerometer is suitable for environments with a short working time and minimal external temperature influence.

## Figures and Tables

**Table 1:** Comparison of data before and after various compensation for an ACC3 accelerometer.

Name	Uncompensated	Voltage Reference Source Compensation	Terminal Temperature Compensation	Combined Compensation
Zero bias variation over temperature (p–p) μg	4098	3367	185	124
Zero bias stability over temperature (1σ) μg	1374	1156	59	40
Scale factor variation over temperature (p–p) ppm	6606	765	83	46
Scale factor stability over temperature (1σ) ppm	2233	242	27	16

## Data Availability

Not applicable.
